# Time- and Behavioral State-Dependent Changes in Posterior Hypothalamic GABA_A_ Receptors Contribute to the Regulation of Sleep

**DOI:** 10.1371/journal.pone.0086545

**Published:** 2014-01-21

**Authors:** Denys V. Volgin, Jackie W. Lu, Georg M. Stettner, Graziella L. Mann, Richard J. Ross, Adrian R. Morrison, Leszek Kubin

**Affiliations:** 1 Department of Animal Biology, School of Veterinary Medicine, University of Pennsylvania, Philadelphia, Pennsylvania, United States of America; 2 Department of Psychiatry, School of Medicine, University of Pennsylvania, Philadelphia, Pennsylvania, United States of America; 3 Behavioral Health Service, Philadelphia Veterans Affairs Medical Center, Philadelphia, Pennsylvania, United States of America; Kent State University, United States of America

## Abstract

Sleep-wake behavior is regulated by a circadian rhythm, homeostatically and by additional mechanisms that determine the timing of slow-wave sleep and rapid eye movement sleep (REMS) episodes. The posterior hypothalamus coordinates the neural and humoral signals with the rest-activity cycle. It contains wake-active neurons, and is a site where stimulation of inhibitory GABA_A_ receptors promotes sleep, whereas their antagonism enhances wakefulness. We explored whether GABAergic mechanisms present in the posterior hypothalamus contribute to the homeostatic and other aspects of sleep-wake regulation. Using micropunches of tissue extracted from either the perifornical (PF) or dorsomedial (DM) regions of the posterior hypothalamus of rats, we determined that mRNA levels for selected subunits of GABA_A_ receptors (β1, β3 and ε) were higher at the end of the active period or following sleep deprivation, when the need for sleep is high, than after several hours of sleep, when sleep need is partially fulfilled. Such a pattern was present in the PF region only, and was consistent with changes in β1 subunit and GABA synthesizing enzyme (GAD) protein levels. In contrast, in the DM region, the levels of GABA_A_ receptor subunit mRNAs and proteins (α1, α2, β1) and GAD varied with circadian time, but were not responsive to sleep deprivation. Separate experiments with sleep-wake monitoring and local perfusion of the PF region with the GABA_A_ receptor antagonist bicuculline revealed that the antagonist had a weaker sleep-reducing effect when sleep need was enhanced by sleep deprivation and that the increased amount of REMS characteristic of the late sleep period was dependent on endogenous GABAergic inhibition. These results support the concept that a varying magnitude of GABAergic inhibition exerted within the PF region contributes to the homeostatic regulation of sleep and shapes its temporal pattern, whereas GABAergic mechanisms in the DM region contribute to circadian regulation.

## Introduction

Sleep-wake behavior is regulated by the circadian rhythm and homeostatic drive, with the strength of the latter increasing with the duration of prior time spent awake [1]–[3]. The cellular and biochemical mechanisms underlying the circadian rhythm of sleep and the main source of these signals, the suprachiasmatic nucleus of the anterior hypothalamus, have been well defined [4]–[6]. In contrast, the neurochemical substrate(s) underlying the homeostatic regulation of sleep are a subject of intense debate and research. There is evidence that accumulation of cellular metabolites, such as adenosine, and biochemical stress-related changes in the endoplasmic reticulum occurring in both neurons and glia during wakefulness are responsible for the buildup of the drive for sleep [7]–[15]. Depletion of energy stores, such as glycogen, is another potential driver of the “sleepiness signal” [16], [17]. It also appears that different brain regions use different neurochemical mechanisms that ensure a cellular rest following a period of cellular activity associated with wakefulness [18]–[20]. For example, growth hormone-releasing hormone, brain-derived neurotrophic factor and cytokines act as sleep-promoting molecules in the cerebral cortex and other brain regions [21]–[24]. Furthermore, each of the two major sleep states, slow-wave sleep (SWS) and rapid eye movement sleep (REMS), has features of a homeostatically regulated process, and a temporal structure, such that the relative amounts of SWS and REMS gradually shift in favor of the latter with the duration of the sleep period [25], [26]. To orchestrate this complex regulation, multiple mechanisms must act both locally and systemically, and their contributions must be coordinated by a neural network to achieve a sleep-wake behavior pattern that optimally meets the diverse needs of the entire organism [27].

The perifornical (PF) region of the posterior hypothalamus plays an important role in the control of sleep and vigilance and coordinates multiple behaviors that are closely linked to the sleep-wake cycle [28], [29]. The region contains several neuronal groups with well-defined phenotypes that contribute to these functions, including the orexins (ORX)- and melanin-concentrating hormone (MCH)-synthesizing neurons [30]–[34]. The region also has dense GABAergic innervation that originates in the anterior hypothalamus, as well as local GABAergic interneurons [35]–[37]. Endogenous GABA release increases in the PF region during SWS [38]. This increase may contribute to the maintenance of sleep because stimulation of GABA_A_ receptors (GABA_A_Rs) located in the PF region with the agonist muscimol promotes SWS [39] and suppresses activity of wake-active neurons [40], whereas local antagonism of these receptors reduces sleep [41], [42].

Importantly, in cats made insomniac by anterior hypothalamic lesions, the sleep-promoting effect of GABAergic inhibition exerted within the posterior hypothalamus was augmented [43]. This observation prompted us to hypothesize that GABA_A_R expression in the PF region may increase with the duration of wakefulness and that this may be an important mechanism underlying increased pressure for sleep. Specifically, it appeared plausible that a prolonged wakefulness could lead to an increased expression (or sensitivity) of GABA_A_Rs located in the PF region. This, in turn, would enhance the strength of GABAergic inhibition at this site and translate into a stronger sleep-promoting signal, increased subjective sleepiness and deeper sleep. We previously tested selected aspects of this hypothesis by superfusing posterior hypothalamic slices *in vitro* with either GABAergic agonists (to mimic a period of increased GABA release during sleep), or a GABA_A_R antagonist (to mimic a state of reduced inhibition) [44]. The results from this reduced model supported the concept that a few hours of increased cellular activity lead to a significant and regionally selective upregulation of mRNA for selected subunits of GABA_A_Rs in the PF region.

Our present goal was to test our hypothesis under the *in vivo* conditions of experimentally varied sleep drive. We quantified selected mRNA and protein levels important for GABAergic transmission in the PF and the adjacent dorsomedial/paraventiricular (DM) region of the posterior hypothalamus. We then functionally tested whether the effects on sleep of pharmacological antagonism of endogenous inhibition mediated within the PF region by GABA_A_Rs vary with the pressure for sleep. We found that the strength of GABAergic inhibition in the PF region increases when the drive for sleep is increased, that changes in GABA_A_R expression likely contribute to this process, and that sleep drive-related changes in the PF inhibition mediated by GABA_A_Rs differentially affect SWS, REMS and motor activity during wakefulness. Preliminary results have been published [45]–[47].

## Materials and Methods

### Ethics Statement

All animal procedures followed the guidelines of the Guide for the Care and Use of Laboratory Animals of the National Institutes of Health and were approved by the Institutional Animal Care and Use Committee of the University of Pennsylvania (Protocols no. 704337 and 803579). All surgical procedures were performed under anesthesia and with continuous monitoring to ensure stable and pain-free conditions. All efforts were made to minimize any animal discomfort.

### Animals

The study was conducted with 61 adult male Sprague-Dawley rats (300–370 g) obtained from Charles River Laboratories (Willmington, MA). The animals were housed on a 12∶12 light/dark schedule with lights on at 7 am and *ad lib* access to food and water. All experimental procedures were conducted during the lights-on period, which for rats is the natural sleep/rest period. The 23 animals used in the mRNA studies and the 28 animals used in the protein expression studies were divided into three groups: (1) the “AM” group was sacrificed at 9 am (n = 8 and 9 for mRNA and protein, respectively); (2) the “PM” group was sacrificed between 3 and 4 pm following undisturbed sleep (n = 8 and 10, respectively); and (3) the “PM-SD” group was sacrificed between 3 and 4 pm following 6 h of sleep deprivation (SD) applied from 9 am to 3 pm (n = 7 and 9, respectively). To maintain an undisturbed social environment, the rats used in these studies were housed in pairs. During SD, both rats were kept in their home cage, with only one animal targeted for SD until the time when it was euthanized prior to the tissue extraction procedure. SD was achieved by presenting the animals with a novel object (e.g., a paper towel) or stroking the targeted animal with a soft brush whenever it assumed a curled posture indicative of intent to enter sleep. Three rats were sacrificed at 3∶00 pm after undisturbed sleep and transcardially perfused; forebrain slices were immunohistochemically stained for glutamic acid decarboxylase (GAD; mouse antibody against both 65 kDa and 67 kDa isoforms, catalog no. M018-3; MBL International, Woburn, MA). An additional 7 rats were instrumented and, after habituation, their sleep-wake behavior was monitored under different conditions of sleep need, with or without perfusion of the PF region of the posterior hypothalamus with the GABA_A_R antagonist, bicuculline.

### Collection and Processing of Tissue Samples for mRNA and Protein Extraction

**Figure 1:**
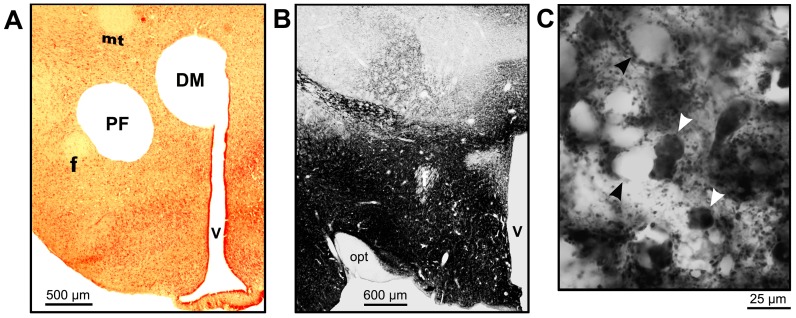
Tissue sampling from the GABA-rich posterior hypothalamic regions to assess their role in sleep regulation. A: Neutral red-stained, coronal section from a hypothalamic slice used to extract tissue micropunches from the perifornical (PF) and dorsomedial (DM) regions. B: low-magnification image of a posterior hypothalamic section immunostained for the GABA-synthesizing enzymes GAD65/67 shows that the entire posterior hypothalamus contains high GAD levels. C: enlarged image of a small area of the PF region demonstrates that GAD staining occurs both in small cell bodies (white arrowheads point to two examples) and in punctae representing axon terminals. Embedded among GAD65/65-containing cells and terminals are large cell bodies that are GAD-negative (black arrowheads point to two examples). Abbreviations: f – fornix, mt – mammillothalamic tract, opt – optic tract, V –3^rd^ ventricle.

Rats were deeply anaesthetized with isoflurane (3.5%) and decapitated. The forebrain was then rapidly removed and immersed in ice-cold medium containing (in mM): 135 NaCl, 5 KCl, 1 CaCl_2_, 1 MgCl_2_, 10 HEPES, 10 glucose, and 20 mannitol; pH 7.4, osmolarity 300±5 mOsm [48]. The hypothalamus was blocked and immersed in the same ice-cold medium and transverse sections, 400 µm-thick, were cut with a tissue slicer (VSLM1; Lafayette, IN) from the region just caudal to the decussation of the optic tract. Two circular tissue punches (700 µm) were extracted. One was taken from the PF region and the other from the more dorsomedially located region that included the dorsomedial hypothalamic nucleus, and the caudal portions of the paramedian nucleus and dorsomedial hypothalamic area, as defined in a rat brain atlas [49]. The latter region is referred to here as the DM region. Figure 1A shows an example of a hypothalamic slice from which tissue punches were extracted, and Fig. 1B shows a low-magnification image of a brain section representing the antero-posterior level similar to that shown in panel A that was immunohistochemically stained with antibodies against GAD65/67. Consistent with earlier reports [35], [50], the posterior hypothalamus contains very high density of GAD65/67, whereas the adjacent fiber tracts (fornix and optic tract), the dorsally located ventromedial and reuniens thalamic nuclei, and parts of the paramedian hypothalamic nucleus contain considerably less staining. The high-magnification image in panel C demonstrates that the high-intensity GAD65/67 staining seen in panel B originates in both terminal fibers and small GABA-ergic cell bodies. These GAD65/67-stained structures surround unidentified large cells.

Protein samples were similarly harvested from the PF and DM regions of the posterior hypothalamus from 500 µm-thick slices. They were frozen on dry ice and stored at −80°C. The slices from which the punches were taken were fixed in 10% paraformaldehyde, cryoprotected in 30% sucrose, cut into 25 µm sections, mounted on slides and stained with Neutral red.

### mRNA and Protein Quantification

The reverse transcription-polymerase chain reaction (RT-PCR) procedures used for mRNA quantification were described previously [44], [48]. Total RNA was extracted from each punch using the RNeasy-mini kit (Qiagen, Valencia, CA), re-dissolved in 50 µl of RNase-free water and quantified by densitometry (BioPhotometer, Eppendorf, Hamburg, Germany). One half of the extract was treated with RNase-free DNase I (Roche Diagnostics, Germany) and reverse-transcribed using PowerScript reverse transcriptase (BD Biosciences Clontech, Palo Alto, CA) in a total buffer volume of 50 µl. Subsequent PCRs were monitored using LightCycler system (Roche Diagnostics, Indianapolis, IN). Fixed aliquots of each cDNA sample (1 µl) were used for PCR with primer sets for selected GABA_A_R subunits, GAD isoforms 65 and 67, prepro-orexin, and α-tubulin (see primer set list in the supplemental information in [44]). The criteria for primer specificity, reaction quality control, and steps to optimize PCR conditions were described previously [51]. PCR amplification was performed in 20 µl of the reaction buffer containing 250 µM of dNTPs, 200 nM of the primers, 2.5 µl of SYBR Green I cDNA-sensitive dye (Sigma-Aldrich, Saint Louis, MO), 1 µl of cDNA sample, and 0.6 µl of Titanium Taq DNA polymerase (BD Biosciences Clontech). The reactions comprised 30 s of initial denaturation at 95°C followed by 20–40 cycles consisting of a 0 s spike at 95°C and 25 s of combined annealing-elongation at 68°C, and were concluded with 30 s of final elongation. Subsequently, the products were melted by linear heating (0.2°C/s) to 95°C. The data inclusion-exclusion criteria were based on histological verification of the punch locations and assessment of RNA/cDNA quality. The yield of the total RNA extracted from a punch had to be 0.1–1.0 µg, the A_260_/A_280_ coefficients ratio within 1.3–2.0, and the A_320_ coefficient less than 0.1. The quality of PCRs was then assessed based on the presence of a single peak on the differentiated cDNA melting curve at a temperature appropriate for the expected product and band position on an ethidium bromide-stained 2% agarose gel. To control for genomic DNA amplification, at least one DNase-treated but not reverse-transcribed RNA sample from each rat was submitted to PCRs with various primers. None of these reactions was positive.

PCRs were calibrated using external cDNA standards obtained by amplification of the target cDNAs synthesized from total rat hypothalamic RNA, purification of the product with a gel extraction kit (QIA-quick; Qiagen), and then densitometric determination of cDNA concentration in the master standard solution. Calibration curves were then generated for each transcript as previously described [52]. The target cDNA species present in individual test samples were quantified as the number of copies in 1 µl of the reverse-transcribed sample per 1 ng of total RNA extracted from that sample.

For protein quantification, each micropunch sample was sonicated in 12 µl of a solubilizing buffer containing 7 M urea, 2 M thiourea, 0.25 mM Tris base, 4.0% CHAPS and 1% NP-40. The soluble fraction of the homogenate was loaded onto precast polyacrylamide Tris-HCl minigels (Ready gel; Bio-Rad, Hercules, CA) in a buffer containing 100 mM Tris-HCl, 200 mM DTT, 4% SDS, 0.2% bromophenol blue and 20% glycerol. Proteins were separated and transferred to nitrocellulose membranes (Mini Protean-3; Bio-Rad). Membranes were blocked for 60 min in Tris-buffered saline (TBS) containing 5.0% non-fat dry milk and 0.1% Tween-20 (pH 7.4) and then incubated overnight in rabbit antibodies against the β1 subunit of the GABA_A_ receptor (1∶1,000, catalog no. OPA1-04106; Pierce/Thermo Scientific, Waltham, MA) and β-actin (1∶1,000, catalog no. 4970; Cell Signaling, Danvers, MA) at 4°C. Primary antibody binding was visualized using donkey enhanced chemiluminescence anti-rabbit horseradish peroxidase-conjugated IgG (1∶10,000, catalog no. NA934V; GE Healthcare/Amersham, Piscataway, NJ) and chemiluminescent substrate (Super Signal West Dura; Pierce/Thermo Scientific). Chemiluminescence was detected using autoradiography film (HyBlot-CL; Denville Scientific, South Plainfield, NJ). Following documentation of β1 subunit immunostaining, the membranes were washed in TBS and stripped of antibodies by incubation in a stripping buffer (Pierce/Thermo Scientific). The membranes were then washed and incubated overnight with mouse anti-GAD antibody against both 65 kDa and 67 kDa isoforms (1∶8,000, catalog no. M018-3; MBL International). They were subsequently incubated with sheep enhanced chemiluminescence anti-mouse horseradish peroxidase-conjugated IgG (1∶5,000, catalog no. NA931V; GE Healthcare/Amersham), and immunostaining was again visualized by chemiluminescence. Films were scanned with 8 bit grayscale resolution, distinct bands were quantified densitometrically (ImageJ software; National Institutes of Health, Bethesda, MD) and, for each sample, the amounts of the β1 subunit and GAD65/67 proteins were expressed relative to the optical density of the band for β-actin.

All mRNA and protein data sets were tested for normality using the Shapiro-Wilk test. The significance of differences between the conditions was examined using one-way ANOVA with Bonferroni correction. When normality criteria were not fulfilled, nonparametric Kruskal-Wallis ANOVA with Bonferroni correction was applied. Differences were considered significant at p<0.05 (Analyse-It statistical software, Leeds, UK). As is customary in sleep and circadian research, mRNA and protein changes were classified as circadian-related when their levels changed with the phase of the circadian cycle but were insensitive to changes in sleep drive, or as sleep-related, if their levels responded to manipulations of the prior amounts of sleep and wakefulness while showing a lesser or no dependence on the circadian time.

### Animal Instrumentation for Monitoring of Sleep-wake Behavior

Seven rats were anesthetized with ketamine (60 mg/kg) and xylazine (7.5 mg/kg), with anesthesia subsequently maintained with isoflurane (0.5–0.75%). Under aseptic conditions, they were implanted for recording of the cortical EEG, hippocampal activity and neck EMG, as described previously [53]. All leads were connected to a mini-socket attached to the skull. A guide tube for insertion of a microperfusion probe (BAS, West Lafayette, IN) was implanted in the posterior hypothalamus aiming to position the tip of the perfusion cannula at 3.0 mm posterior to bregma, 1.2 mm to the left of the midline, and 8.6 mm below the cortical surface. At the end of instrumentation surgery, yohimbine (5.0 mg/kg), gentamicin (5.0 mg/kg) and butorphanol (2.0 mg/kg) were administered to accelerate the recovery from anesthesia, to protect the animal from infection, and for analgesia, respectively. The rats were then allowed at least a 7-day recovery period during which they were housed individually with lights on from 7 am to 7 pm and with free access to food and water. Subsequently, each animal was transferred in its home cage at least three times for 1–4 h/day to a dimly illuminated and sound-attenuated chamber for habituation to the recording conditions, first without and then with the recording cable attached and with a microdialysis cannula inserted and perfused with artificial cerebrospinal fluid (acsf). The acsf composition was (in mM): 147 NaCl, 4 KCl, 2.3 CaCl_2_, 1.9 MgCl_2_.

### Sleep-wake and Motor Activity Recording and Hypothalamic Microperfusion Protocol

All signals were amplified (P511 amplifiers; Grass Instruments, West Warwick, RI) and digitally acquired using Spike 2 software (CED, Cambridge, UK) at a rate of 100 Hz for the EEG and hippocampal activity, and 1000 Hz for the EMG. All recordings lasted 5 h and included continuous perfusion of the PF region with either acsf or the GABA_A_ receptor antagonist bicuculline methiodide (20 µM in acsf, referred to as BIC). Individual recordings sessions were conducted either between 8∶30 am and 13∶30 pm (AM sessions) or between 11∶30 pm and 4∶30 pm (PM sessions). Prior to selected PM sessions, the animals were subjected to SD from 8∶00 am to noon; SD was continued during the first 30 min after the animal was connected to the recording apparatus. While subjected to SD, the animal remained in its home cage and was gently prompted to maintain wakefulness in the same way as with the animals designated for tissue extractions. Motor activity was quantified in successive 10 min intervals using an infrared beam crossings technique, with the beams spaced at 1 inch in the horizontal plane and each crossing counted as a separate event (AccuScan Instruments, Columbus, OH).

Hypothalamic microperfusion was conducted using a cannula with a 1 mm-long, semi-permeable membrane (CMA-11; BAS, West Lafayette, IN). A continuous flow of either acsf or BIC at a rate of 0.8 µl/min was maintained throughout each recording session (MD-1001; BAS). Each animal was subjected to six time-of-the-day/drug/handling conditions (AM-acsf, AM-BIC, PM-acsf, PM-BIC, PM-SD-acsf, PM-SD-BIC) that were applied in a random order and separated by at least two days. During the AM sessions with hypothalamic perfusion with BIC, the probe was initially perfused with acsf and then, using a liquid switch (CMA-110; BAS), BIC flow was switched on at 9∶30 am and switched back to acsf at 12∶30 pm. Similarly, during the PM sessions with BIC, the probe was initially perfused with acsf, and then BIC flow was switched on at 12∶30 pm and switched back to acsf at 3∶30 pm; these times represent the times when the new solution reached the brain after accounting for the delay in the perfusion system. Sufficiently high concentrations of bicuculline perfused into the PF region can strongly suppress sleep and elicit a high level of motor activity [41], [54]. To avoid excessive motor activation that would then disrupt sleep, we chose to use in the present experiments a relatively low concentration of bicuculline that was sufficient to moderately affect sleep-wake amounts but did not produce exaggerated motor activity. At the selected concentration (20 µM), bicuculline at least partially antagonized the endogenous effects of GABA on GABA_A_Rs within a limited region surrounding the tip of the perfusion probe.

At the end of the study, the animals were deeply anesthetized with pentobarbital sodium (100 mg/kg, i.p.) and were transcardially perfused with phosphate-buffered saline followed by 4% paraformaldehyde. The forebrains were cryoprotected in 30% sucrose, sectioned in the coronal plane into 35 µm sections, serially mounted, and stained with Neutral red to verify the location of the perfusion site.

### Sleep Scoring and Behavioral Data Analysis

Sleep-wake states were scored in 10 s epochs as wakefulness, SWS and REMS on the basis of the appearance of the cortical, hippocampal and EMG signals, with the scoring process assisted by simultaneous display of signal powers in selected frequency bands (Somnologica; Medcare, Buffalo, NY), as described previously [53], [55]. Statistical analysis was conducted within subjects using repeated measures, two-way ANOVA followed by paired Student’s t-tests.

## Results

### GABA_A_ Receptor Subunit and GABA Synthesizing Enzyme, GAD, mRNA Levels Vary in the Posterior Hypothalamus with Sleep Need and Circadian Time

**Figure 2:**
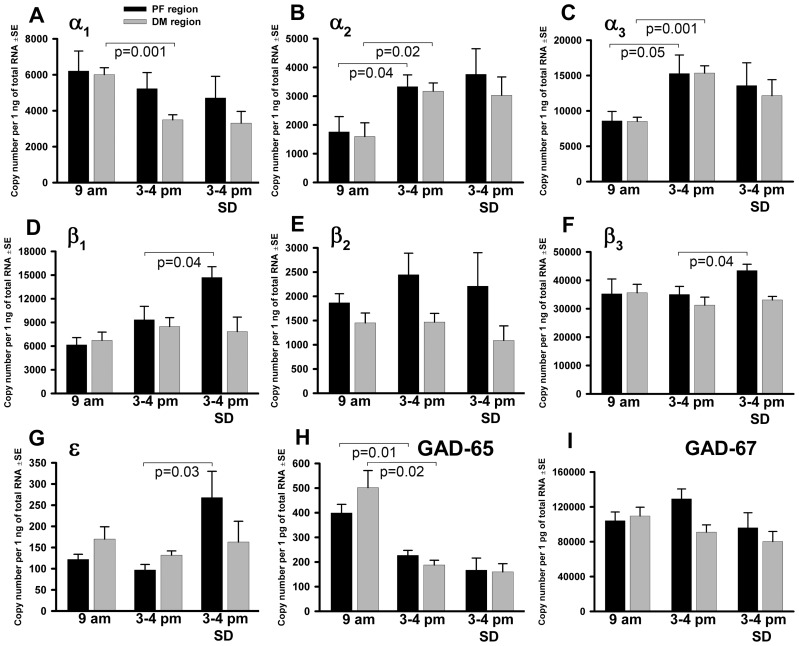
GABA_A_ receptor subunit and GAD mRNA levels in the perifornical (PF) and dorsomedial (DM) regions of the posterior hypothalamus in relation to sleep need and circadian time. Quantification was conducted using tissue micropunches extracted from the two regions from animals sacrificed at 9–4 pm following undisturbed sleep, and at 3–4 pm following sleep deprivation (SD). Significant differences between the 9 am and 3–4 pm groups that were not modified by SD were detected for the α2 and α3 subunits of GABA_A_ receptor and for the GAD65 isoform in both regions, and for the α1 subunit in the DM region. When the effects of time of the day were not altered by SD, they were categorized as circadian-dependent. In contrast, mRNA levels for β1, β3 and ε subunits of the GABA_A_ receptor, did not differ between 9 am and 3–4 pm groups, but were significantly increased in the 3–4 pm group subjected to SD. Such sleep need-dependent changes occurred in the PF region only. N = 6–8 rats per group.

We quantified the effects of circadian time and sleep drive on mRNA levels for most GABA_A_R subunits present in the posterior, lateral hypothalamus [56]–[59], GABA synthesizing enzymes and prepro-ORX. The level of α1 subunit mRNA was significantly higher at 9 am than at 3–4 pm in the DM, but not the PF, region (Fig. 2A), whereas the mRNA levels for α2 and α3 subunits were significantly lower at 9 am than at 3–4 pm in both regions (Fig. 2B and C). SD did not significantly affect any of these changes, as indicated by the absence of a difference between the 3–4 pm group that was allowed to sleep *ad lib* and the SD group.

In contrast to α subunits, the mRNA levels of β1, β3 and ε subunits did not significantly differ between the 9 am and 3–4 pm groups when compared under the conditions of undisturbed sleep, but they were significantly increased in the 3–4 pm group subjected to SD when compared to the same circadian time after undisturbed sleep. Also in contrast to α subunits, the increases occurred in the PF region only, whereas in the DM region there was no evidence of an effect of either time of day or SD on these subunits (Figs. 2D, 2F and 2G). The β2 subunit mRNA did not exhibit any significant differences among the three experimental groups (Fig. 2E).

Table 1 shows numerical data for measurements of GABA_A_R subunit mRNA levels in the PF and DM regions depicted in Fig. 2 and categorizes them according to their dependence on circadian period and sleep drive, as defined in the Methods. Three subunits exhibited significant sensitivity to SD (β1, β3 and ε), and none of these exhibited any significant variation with circadian time. Notably, in all three cases, the sleep drive-dependence was limited to the PF region. In contrast, circadian dependence was more common and less region-specific. The three α-type subunits had significant circadian time-related changes. Of these, α2 and α3 subunit mRNAs had significantly elevated levels during the late part of the rest period and changed in the same direction in both the PF and DM regions, whereas α1 subunit mRNA was lower during the late part of the rest period and the change was significant only in the DM region.

**Table 1:** Mean mRNA levels of GABA_A_ receptor subunits, GABA-synthesizing enzymes GAD65 and 67, and prepro-orexin (ORX) in relation to sleep need and circadian time in the posterior hypothalamic perifornical (PF) and dorsomedial (DM) regions.

	mRNA levels^a^ in the PF region at different circadian times and sleep need condition				mRNA levels^a^ in the DM region at different circadian times and sleep need condition			
	9 AM	3–4 PM	3–4 PMafter SD	Biologicalrhythm effect	9 AM	3–4 PM	3–4 PMafter SD	Biological rhythmeffect
**GABA_A_** **α1**	6,200±1,100(n = 7)	5,220±900(n = 8)	4,710±1,200(n = 6)	**None**	6,010±380(n = 6)	3,490±280^AM^(n = 7)	3,310±650(n = 7)	**Circadian**
**GABA_A_** **α2**	1,760±530(n = 7)	3,330±410**^AM^**(n = 8)	3,760±890(n = 7)	**Circadian**	1,590±480(n = 8)	3,170±290**^AM^**(n = 8)	3,000±640(n = 7)	**Circadian**
**GABA_A_** **α3**	8,600±1,300(n = 7)	15,000±2,400^AM^(n = 8)	13,600±3,200(n = 7)	**Circadian**	8,510±590(n = 6)	15,400±1,300**^AM^**(n = 6)	12,200±2,300(n = 6)	**Circadian**
**GABA_A_** **β1**	6,160±920(n = 7)	9,300±1,700**^SD^**(n = 8)	14,700±1,400(n = 6)	**Sleep**	6,700±1,000(n = 7)	8,500±1,100(n = 8)	7,840±1,830(n = 7)	**None**
**GABA_A_** **β2**	1,870±190(n = 6)	2,450±450(n = 8)	2,210±690(n = 6)	**None**	1,450±210(n = 6)	1,470±180(n = 7)	1,090±300(n = 6)	**None**
**GABA_A_** **β3**	35,300±5,200(n = 7)	35,000±2,900**^SD^**(n = 7)	43,400±2,200(n = 7)	**Sleep**	35,600±3,000(n = 7)	31,300±2,800(n = 7)	33,100±1,230(n = 6)	**None**
**GABA_A_** **ε**	122±12(n = 6)	97±13**^SD^**(n = 6)	268±62(n = 6)	**Sleep**	170±29(n = 6)	132±10(n = 6)	163±49(n = 6)	**None**
**GAD65**	399,000±35,000(n = 6)	226,000±20,000**^AM^**(n = 6)	167,000±49,000 (n = 6)	**Circadian**	502,000±69,000(n = 6)	188,000±19,000**^AM^**(n = 6)	160,000±33,000(n = 6)	**Circadian**
**GAD67**	1.0**×**10^8^±1.0**×**10^7^(n = 6)	1.2**×**10^8^±1.5**×**10^7^(n = 6)	9.6**×**10^7^±1.7**×**10^7^(n = 6)	**None**	1.1**×**10^8^±1.0**×**10^7^(n = 6)	9.1**×**10^7^±8.4**×**10^6^(n = 6)	8.0**×**10^7^±1.2**×**10^7^(n = 6)	**None**
**Prepro-ORX**	43,600±10,000(n = 7)	17,400±2,800**^AM^**(n = 8)	14,300±2,900(n = 6)	**Circadian**	11,400±3,000(n = 7)	9,700±3,100(n = 8)	6,000±1,300(n = 7)	**None**

a – mRNA levels are expressed as copy numbers per 1 ng of total RNA in tissue sample ± standard error; ^AM^ – significantly different from the 9 AM group; ^SD^ – significantly different from the 3–4 PM group subjected to sleep deprivation (SD) prior to tissue extraction; numbers in parentheses indicate the numbers of animals used to obtain tissue samples under each condition. See Methods for the criteria used to categorize the effects as either sleep need- or circadian time-dependent.

In addition to the regionally and behaviorally distinct changes detected for different GABA_A_R subunit mRNAs, GAD65 and prepro-ORX mRNA levels were all lower, or tended to be lower, in both regions at 3–4 pm when compared to 9 am. The difference for GAD65 mRNA was significant for both regions (Fig. 2H, Table 1). Notably, according to our classification, all these changes were circadian time-, rather than sleep need-, related and they did not exhibit any regional selectivity. The circadian dependence of prepro-ORX mRNA was consistent with previous reports [60]–[62], with the effect not being statistically significant in the DM region probably due to few ORX cells extending this far from the PF region [63]. No significant differences were detected among the three conditions for GAD67 mRNA in either region (Fig. 2I and Table 1).

### GABA_A_ Receptor β1 Subunit and GAD Protein Levels are Elevated in the Hypothalamic PF Region when Sleep Need is Increased

**Figure 3:**
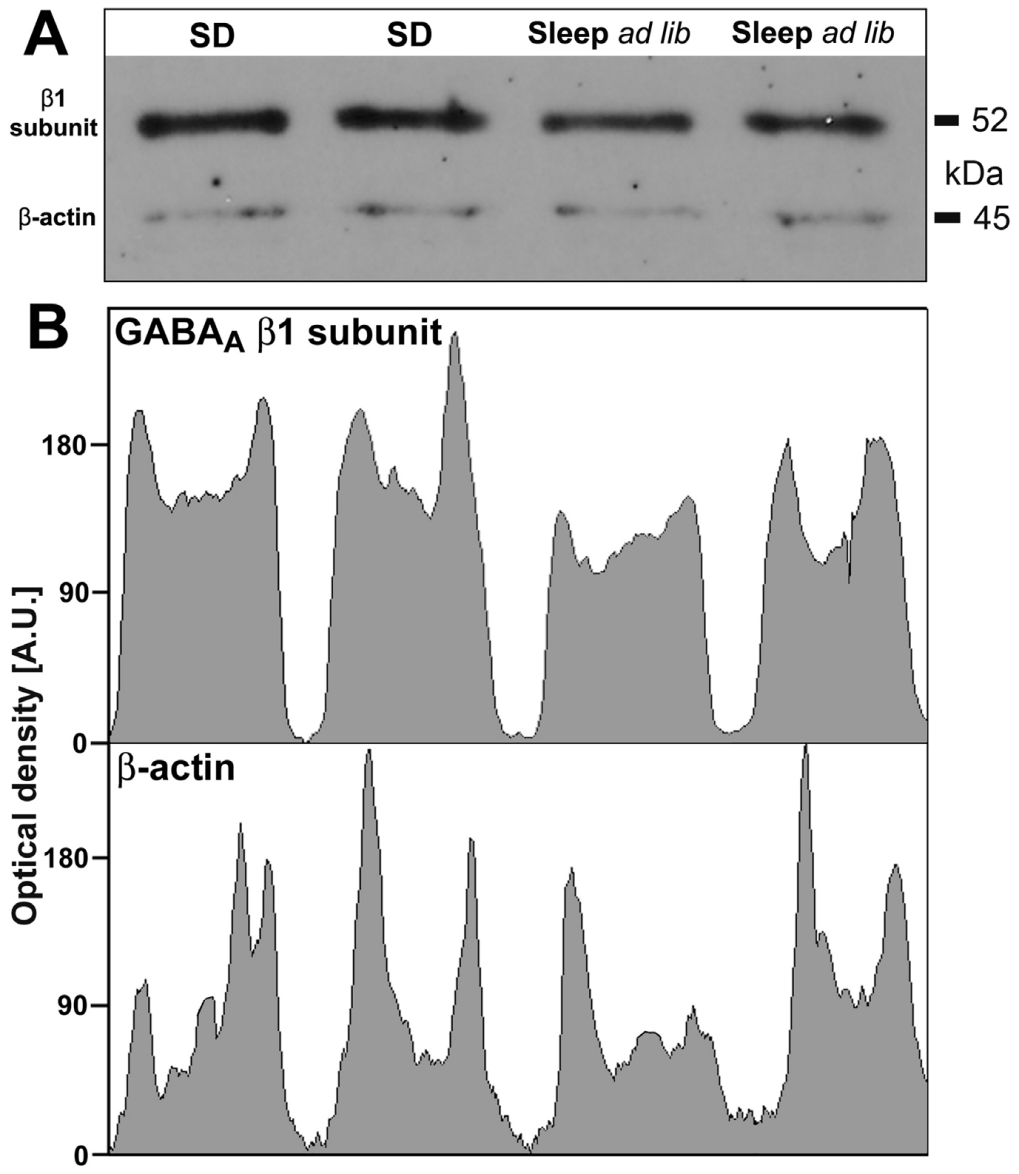
Example of a Western blot with proteins extracted from tissue punches from the perifornical hypothalamic region from two rats subjected to sleep deprivation (SD) for 6 h before they were sacrificed at 3–4 pm and from two rats that slept *ad lib* before they were sacrificed also at 3–4 pm. A: image of the blot. B: scans of optical density across the blot shown in A conducted separately for the β1 subunit of GABA_A_ receptor (top) and β-actin (bottom), with background subtracted. The ratio of the mean optical densities (β1 subunit/β-actin) was used to quantify the effect of SD on the level of β1 subunit protein. In this example, the ratio was 1.57–1.6 for the two rats subjected to SD, and 1.2–1.34 for the two rats that slept *ad lib* prior to sample collection.

Although antibodies for many GABA_A_R subunits are available [56]–[58], [64], we were able to establish a satisfactorily sensitive and specific assay for quantification of protein levels in a small brain region for only one subunit of those that varied with sleep need, the β1 subtype. We also quantified the combined levels of GAD65/67 proteins using antibodies that recognized both GAD isoforms. Figure 3A shows an image of a transfer membrane stained for β1 subunit and β-actin proteins with tissue samples extracted from the PF region at 4 pm for two rats subjected to SD and another two rats whose sleep was undisturbed until 4 pm. Panel B shows the distribution of staining intensity for the β1 subunit (top) and β-actin (bottom) within each lane of the gel shown in A.

**Figure 4:**
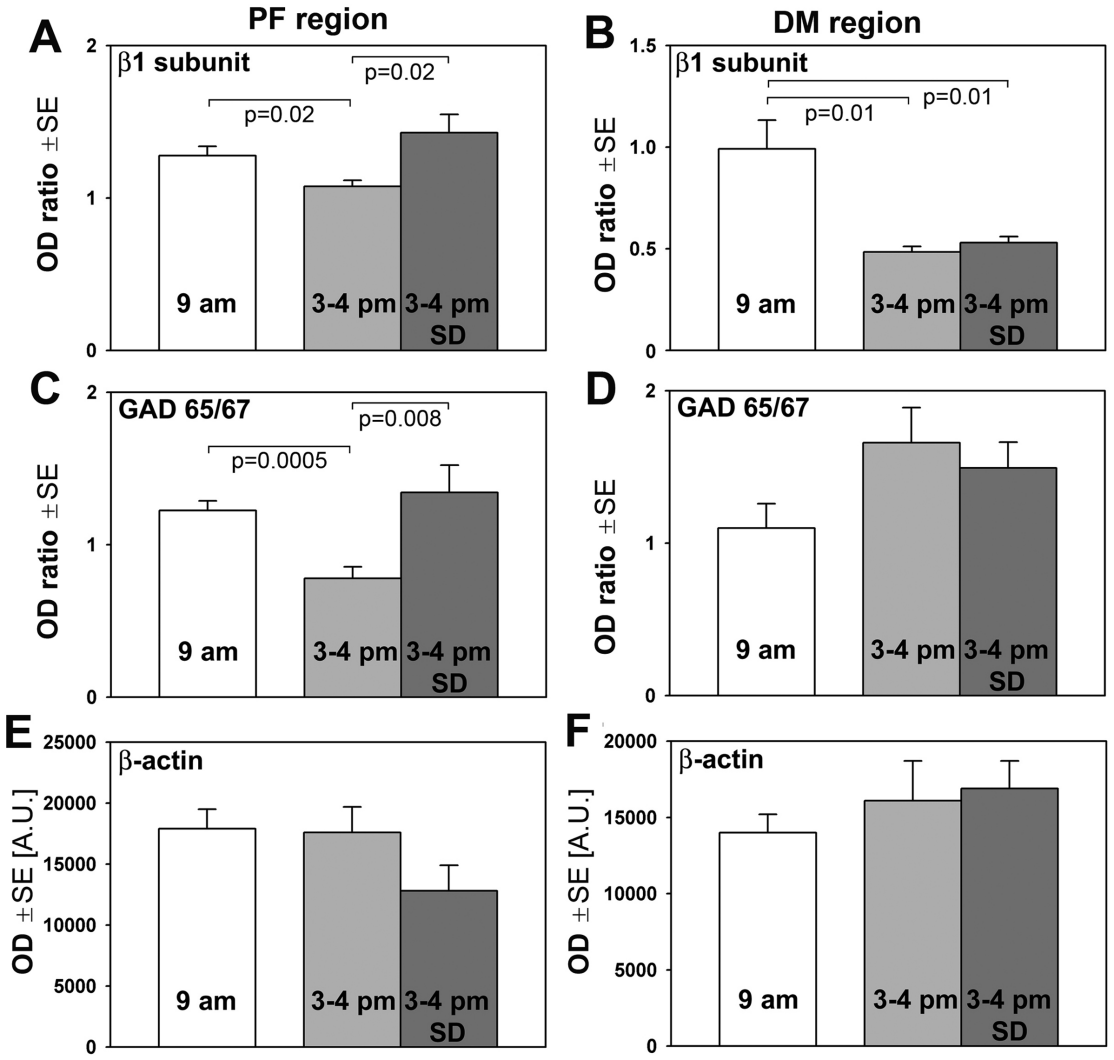
The levels of the β1 subunit of GABA_A_ receptor and GAD65/67 proteins in the perifornical (PF) and dorsomedial (DM) posterior hypothalamic regions in relation to sleep need and circadian time. A: β1 subunit protein level was significantly higher in the PF region in the 3–4 pm group subjected to sleep deprivation (SD) than in the 3–4 pm group sleeping *ad lib* prior to tissue extraction. This was consistent with the findings for the β1 subunit mRNA in this region (Fig. 2D). In addition, the β1 subunit protein level was significantly lower in the 3–4 pm group sleeping *ad lib* when compared to the 9 am group. These changes could be driven by varying sleep need; lower at 3–4 pm than at 9 am but increased relative to the 9 am level by SD. B: in the DM region, β1 subunit protein levels were lower in both 3–4 pm groups when compared to the 9 am group, with no effect of SD. C: in the PF region, quantification of GAD65/67 protein levels revealed a similar pattern of changes as those for the β1 subunit in this region and a similar decrease in the 3–4 pm group when compared to the 9 am group as that found for GAD65 mRNA (Fig. 2H). D: in the DM region, no significant differences were detected among the three groups for GAD65/67 protein, with the trends being unlike those found for either GAD65 or GAD67 mRNA in this region (Fig. 2H and I). E and F: the corresponding mean absolute optical densities (ODs) for β-actin did not exhibit any significant time- or sleep need-dependence, indicating that β-actin levels *per se* were not the main source of the effects on β1 subunit and GAD65/67 proteins. N = 7–9 rats per group.

Figure 4 summarizes the protein quantification results from three rat groups comparable to those used in the mRNA quantification protocol. We found that the ratio of β1 subunit to β-actin protein levels was higher at 9 am than at 3–4 pm in both regions (1.28±0.06 *vs*. 1.08±0.04 in the PF region, and 0.99±0.14 *vs*. 0.48±0.03 in the DM region). In contrast, following SD, the β1 subunit protein level significantly increased in the PF, but not the DM, region when compared to the 3–4 pm group with undisturbed sleep (to 1.43±0.12; Figs. 4A and B). Thus, the effect of SD on the β1 subunit protein level in the PF region was consistent with that found in this region for this subunit mRNA. In addition, protein measurements revealed an effect of circadian time on this subunit that was more prominent in the DM, than the PF, region.

The levels of GAD protein were significantly lower in the PF region at 3–4 pm than at 9 am in the undisturbed sleep condition, but SD eliminated this decline (1.22±0.06 at 9 am, 0.78±0.08 at 3–4 pm, and 1.34±0.18 at 3–4 pm following SD; Fig. 4C). In contrast, in the DM region, GAD protein levels did not differ among the three experimental conditions (Fig. 4D). To verify that the differences in the relative amounts of the target proteins were not secondary to the sleep need- and/or time-dependent changes in β-actin level, we compared the mean absolute values of immunostaining for β-actin among the three study groups. These measurements revealed no significant differences (Fig. 4E and F).

### Effects of GABA_A_ Receptor Antagonism in the Hypothalamic PF Region on Sleep Vary with Sleep Need and Circadian Time

**Figure 5:**
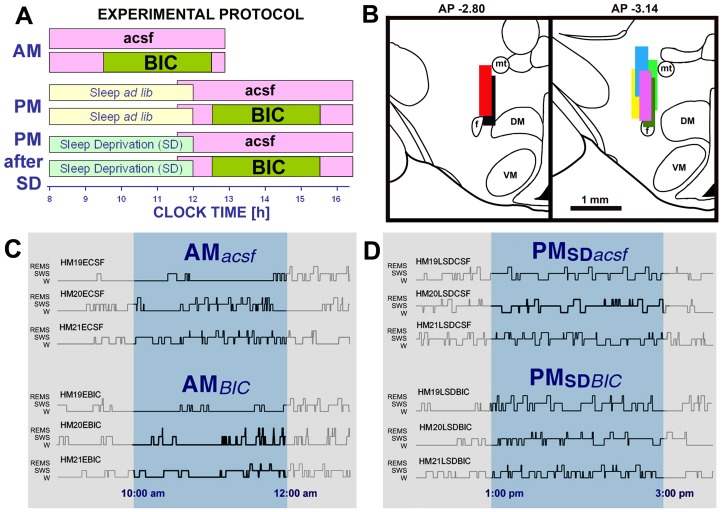
Experimental protocol and data from individual experiments with perfusion of the perifornical (PF) region of the posterior hypothalamus with artificial cerebrospinal fluid (acsf) or the GABA_A_ receptor antagonist, bicuculline (BIC) at different circadian times and under normal or enhanced sleep need. A: sleep-wake behavior of each animal was monitored for 5 h on six different days under six different conditions applied in a random order. The conditions were: recording during the morning with the PF region continuously perfused with acsf only or with BIC substituted for acsf during the middle part of the recording (AM sessions); recording during the afternoon with the PF region continuously perfused with acsf only or with BIC for 3 h during the middle part of the recording (PM sessions); and recording during the afternoon preceded by sleep deprivation (SD) applied from 8 am through noon (PM sessions with SD) with the PF region continuously perfused with acsf only or with BIC for 3 h during the middle part of the recording. B: locations of the active portions of the perfusion probes, as determined after conclusion of each study, superimposed on the closest standard coronal sections of the posterior hypothalamus derived from a rat brain atlas [49]. Different colors mark probes in different animals. Abbreviations: DM – dorsomedial hypothalamic region, f – fornix, mt – mammillothalamic tract, VM – ventromedial hypothalamic nucleus. C and D: hypnograms from AM and PM recording sessions with either acsf or BIC perfusion of the PF region from three animals. The highlighted portions represent the 2 h periods when BIC concentration around the perfusion site would have achieved a steady state after the switch to perfusion with the antagonist. Perfusion with BIC moderately suppressed sleep, with the effect being more prominent during the AM sessions (C) than during the PM sessions conducted after SD (D).

The results with mRNA and protein measurements supported the concept that sleep need- and circadian cycle-dependent changes in the availability of GABA_A_ receptors in the hypothalamic PF region may contribute to the regulation of sleep. To assess this hypothesis at the functional level, we combined recording of sleep-wake behavior in chronically instrumented rats with local microperfusion of the PF region with acsf or BIC applied at two different circadian times, with or without prior SD. Figure 5A shows the scheme of the six recording conditions used in these experiments.

Figure 5B shows the locations of the active portions of the perfusion cannulae in the posterior hypothalamus. In all 7 rats, the cannulae were located in the PF region, just dorsal to the fornix. Figures 5C and D show examples of the hypnograms from three rats obtained under four different experimental conditions (AM recordings with either acsf or BIC perfusion in C, and PM recordings conducted following SD with either acsf or BIC perfusion in D). They illustrate a moderate sleep-reducing effect of BIC during the AM recordings and moderately increased sleep amounts with a less prominent effect of BIC in the PM recordings conducted after SD.

**Figure 6:**
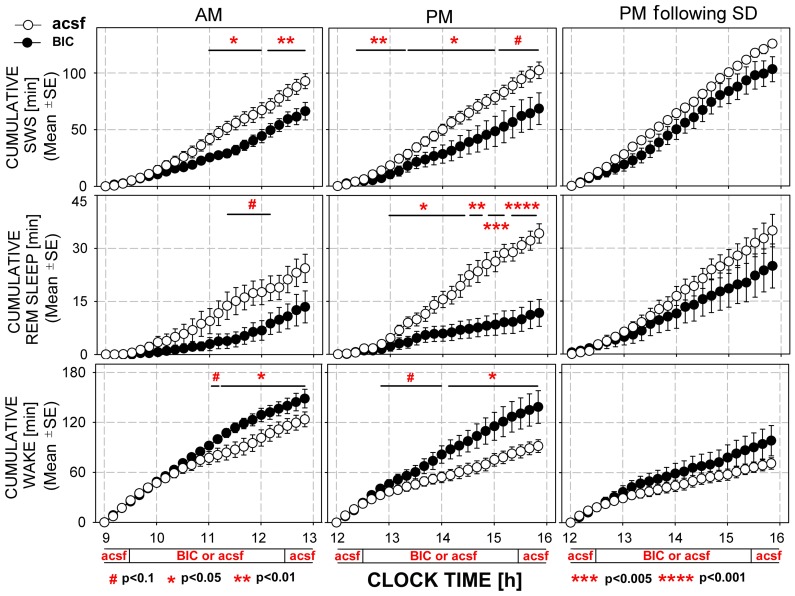
Perfusion of the perifornical (PF) region of the posterior hypothalamus with the GABA_A_ receptor antagonist, bicuculline (BIC) reduced the amounts of slow-wave sleep (SWS) and REM sleep during the early (AM) and late (PM) part of the day, with the reduction of REM sleep being particularly strong during the PM period. The graphs show the cumulative amounts of sleep-wake states in 7 rats studied under the experimental conditions defined in Fig. 5A. Symbols marking different levels of statistical significance refer to paired comparisons between perfusions of the PF region with acsf and BIC at successive time points. Perfusion with BIC during PM recording sessions conducted after sleep deprivation (SD) caused only a trend towards reduced sleep amounts (right column).

Figure 6 compares the cumulative amounts of SWS, REMS and wakefulness generated during the AM and PM recording sessions with and without prior SD when the PF region was perfused with either acsf or BIC. Both SWS and REMS amounts were significantly reduced by BIC during both AM and PM recordings, but the effect of BIC was not significant during the PM recordings preceded by SD. The effects of BIC on REMS amounts were particularly pronounced during the PM recordings. During this period, as expected, the amount of REMS generated during acsf perfusion was significantly higher than over the same period during the AM sessions, reflecting the characteristic increase of REMS during the second half of the rest period [25], [26]. However, this increase was entirely eliminated by perfusion of BIC into the PF region during the PM sessions. Thus, antagonism of GABA_A_Rs in the PF region revealed that endogenous activation of these receptors strongly contributes to the generation of REMS during the second half of the sleep period.

**Figure 7:**
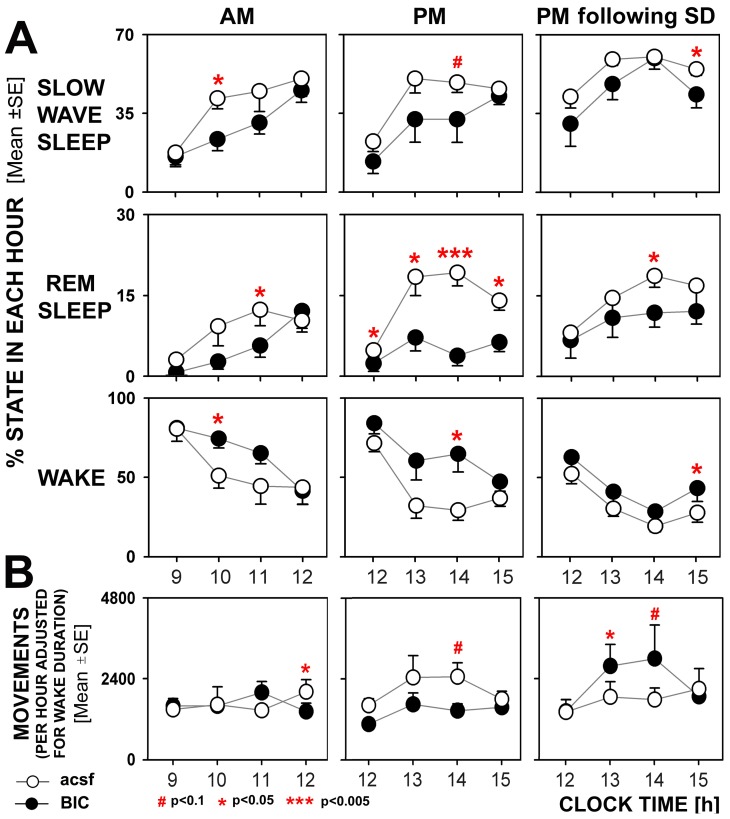
Hourly percentage amounts of sleep-wake states and motor activity during the recording session conducted at different times of day and different levels of sleep drive. A: antagonism of GABA_A_ receptors in the hypothalamic PF region moderately reduced the amount of SWS when applied both at the beginning and in the second half of the rest period (AM and PM recording sessions, respectively), and particularly strongly suppressed the amount of REM sleep during the second half of the sleep period when the amount of this stage of sleep is normally increased. Following sleep deprivation (SD), the effects of bicuculline (BIC) perfusion on sleep were diminished. For the curves marked “BIC,” perfusion with the antagonist was fully established during the two middle intervals, whereas the first and last time points represent the hourly periods during which BIC flow was started and terminated, respectively. In all recording sessions, the effects of BIC waned after termination of the perfusion with the antagonist and most variables returned to the levels recording during continuous perfusion with artificial cerebrospinal fluid (acsf) only. B: hourly counts of movements weighted by the hourly amount of wakefulness. BIC had small and inconsistent effects on motor activity, indicating that the changes in sleep-wake states were not secondary to excessive motor activation. Data from 7 rats; marks for statistical significance refer to paired comparisons between acsf and BIC perfusion session at each interval.

Figure 7A shows the hourly percentage amounts of different sleep wake states corresponding to the cumulative plots shown in Fig. 6. These plots expand on the observations illustrated in Fig. 6 by showing that the effects of BIC were mainly confined to the 3 h periods when the drug was perfused into the PF region and waned after BIC perfusion was switched back to acsf. They also show that, while the cumulative amounts of SWS and REMS were not significantly affected by BIC in the recording sessions conducted following SD (Fig. 6), BIC had moderate but significant sleep-reducing and wake-enhancing effects when quantified on an hourly basis.

Figure 7B shows the hourly amounts of motor activity corresponding to the sleep-wake amounts shown in panel A. The hourly counts of beam crossings are scaled by the percentage amounts of wakefulness on a subject-by-subject and session-by-session basis to eliminate the effect of varying amounts of sleep on quantification of motor activity. Of note here is that, at the concentration used, the effects of BIC on motor activity were modest and not obviously stimulatory. Indeed, only in the PM recording sessions conducted after SD was there a statistically significant (p<0.05) increase of motor activity during the first hour of BIC perfusion and a trend in the same direction in the second hour. Thus, the significant effects of BIC on sleep-wake behavior (panel A) were not secondary to stimulation of motor activity.

## Discussion

A direct comparison of the pattern of changes in mRNA and protein levels for selected GABA_A_R subunits and GAD in two adjacent posterior hypothalamic regions revealed that cells in the wake-promoting (PF) region contain GABA_A_R in which some subunits increase with increased need for sleep and decrease when sleep need is partially discharged. In contrast, in the more dorsomedially located posterior hypothalamic region (DM), selected GABA_A_R subunits varied with circadian time, but not with sleep need when it was increased by SD. Furthermore, the mRNA levels for the GABA synthesizing enzymes, GAD65 and 67, varied in both the PF and DM regions with circadian time, but the combined GAD65/67 protein level increased also in response to SD only in the PF region. Consistent with mRNA and protein quantification results, we found that perfusion of the PF region with an antagonist of GABA_A_Rs significantly reduced sleep under normal sleep conditions but was less effective when sleep need was enhanced by SD. Lastly, we found that inhibition mediated by GABA_A_Rs within the PF region contributes to the progressive increase in the amount of REMS over the duration of a sleep period. Collectively, these results identify dynamic changes in GABAergic mechanisms residing in the PF region of the posterior hypothalamus as potentially important for the homeostatic and time-dependent regulation of sleep and its sub-stages, and GABAergic mechanisms in the DM region of the posterior hypothalamus as important for circadian regulation. In addition, our study specifically suggests that GABA_A_Rs containing β1, β3 and ε subunits located in the PF region contribute to sleep regulatory functions.

We designed our study around the comparison between the PF and DM regions of the posterior hypothalamus because the two regions are adjacent to each other and influence a wide range of fundamental regulatory processes that impact the entire organism but have different afferent and efferent connections and different compositions of prevailing cell phenotypes. The PF region receives projections from sleep-active, anterior hypothalamic GABAergic neurons [36], [37] and contains at least two neuronal groups, ORX and MCH, with established roles in the control of sleep-wake states [31], [33], [34]. The main functions of the DM region relate to neuroendocrine and autonomic homeostasis, including the response to stress [65], and also to the circadian control of sleep and other functions [66]. The principal regional distinction uncovered by our study, that sleep need-related changes are restricted to the PF region, whereas only circadian changes occur in the DM region further highlights the functional difference between the two regions.

We found an upregulation of mRNA (β1, β3, and ε subunits) and protein (β1 subunit and GAD65/67) following SD in the PF region only. Circadian time-related, but sleep need- independent, molecular variations occurred to varying degree in both the PF and DM regions and involved mRNA levels for α1, α2 and α3 subunits of GABA_A_R, as well as GAD65 and prepro-ORX. Thus, in addition to a clear prevalence of sleep need-sensitive effects in the PF region, certain cells and certain types of GABA_A_Rs in both regions participate in circadian regulation. Our data do not provide information about the neurochemical nature of the cells in which changes in GABA_A_Rs and GAD occurred, but studies of the distribution of individual GABA_A_R subunits among hypothalamic cells of different phenotypes offer some guidance. Both ORX and MCH cells express the ε subunit [57]. The α2 subunit is present in hypothalamic MCH neurons, whereas the α3 subunit occurs in ORX neurons [58]. While both ORX and MCH neurons are primarily located in the PF region, their roles in sleep appear to be opposite. ORX neurons are active during wakefulness and exogenous administration of ORX promotes wakefulness [e.g., 32,67–71]. In contrast, activation of MCH neurons, which also contain GABA [33], promotes REMS, SWS, or both [33], [72]–[75]. The PF region also contains a significant contingent of GABAergic neurons that are maximally activated during SWS or REMS [35]. It is likely that some changes in GABA_A_R subunits and GAD that we found occurred in ORX, MCH and GABAergic neurons located in the PF region, but additional studies at the single cell level are needed to unequivocally associate the PF and DM cells in which GABA_A_R expression and/or composition distinctly vary with the need for sleep or circadian time.

GABA_A_ receptors are pentameric ion channels typically composed of α, β, γ, δ and/or ε subunits, with a total of 19 different subunit types and subtypes available for building receptors with different sensitivities to various modulators (e.g., hypnotics, anesthetics, alcohol) and different dynamic properties [76]. As a result of this diversity, different brain regions, nuclei and cell types express GABA_A_Rs with different properties and subunit compositions [40], [77]–[80]. The ability to selectively target GABA_A_Rs with different subunit compositions, albeit currently limited, is important for designing improved therapeutic interventions for sleep disorders [81]–[84]. Within the context of our study, it is of note that mice lacking the GABA_A_R β3 subunit had a normal amount of SWS but a reduced REMS amount during the rest period while their circadian rhythm remained intact for many days even when they were housed under constant dark conditions [85]. In another study, the sleep-promoting effects of oleamide, an endogenous unsaturated fatty acid amide, was abolished in mice lacking the GABA_A_R β3 subunit [86]. The response to SD was not tested in these studies, but a screen of mutations of the α1, β3 and γ2 subunit genes in a human cohort revealed that a missense mutation of the β3 subunit was associated with chronic insomnia [87]. Furthermore, the β3 subunit has been implicated in the pathophysiology of the Angelman syndrome, which includes hyperactivity and a disturbed rest-activity cycle [88]. These findings contrast, for example, with the normal sleep homeostasis in GABA_A_R α3 subunit-knockout mice [89]. It is also of note that, in contrast to distinct changes in sleep amounts and sleep homeostasis in various rodent models with genetically altered expression of GABA_A_ receptors, genetic removal of type B GABA receptors from ORX neurons resulted in fragmentation of sleep-wake episodes but the total amounts of sleep and its circadian pattern were not affected [90]. Collectively, these results demonstrate the importance of GABA_A_Rs, and the β3 subunit in particular, for the regulation of both SWS and REMS sleep, but they do not provide information about the anatomic and cellular location of the relevant GABA_A_Rs. In addition to our data pointing to the PF region of the posterior hypothalamus, an increased immunostaining for the β2/3 subunits of GABA_A_R was found following SD on neuronal membranes of basal forebrain cholinergic neurons [64]. Thus, the regulation of β3 subunit levels in multiple brain regions may contribute to the regulation of sleep.

In addition to the GABA_A_R containing the β3 subunit, GABA_A_Rs located in the PF region and containing the β1 and ε subunits also responded to increased need for sleep. The relevance of our finding that two such subunits were of the β type may be related to the distinct role of these subunits in the assembly of new functional GABA_A_Rs, including their phosphorylation and trafficking to the cell surface [91]–[94].

It has been suggested that, compared to the GAD67, GAD65 levels undergo more rapid changes in response to a varying demand for GABA as a transmitter [95]. Our finding that the GAD65 mRNA declined with the duration of the rest/sleep period suggests that the need for GABA synthesis in the posterior hypothalamus gradually decreases with the duration of sleep as it is maintained by GABAergic inhibition [38], [96]–[98]. Since this effect occurred similarly in the PF and DM regions and was not affected by SD, it may have represented a circadian, rather than sleep need-related, process driving GABA synthesis. However, our combined quantification of both GAD proteins yielded a pattern that was different from that for either the GAD65 or GAD67 mRNA measured separately. The combined GAD protein level declined with the duration of sleep in the PF region, with no significant changes in the DM region. In addition, the GAD65/67 protein level increased in the PF region following SD, whereas there was no effect of SD in the DM region. It is likely that the partial dissociation between GAD mRNA and protein results was related to a relatively lower GAD65 protein levels when compared to GAD67. Since we used antibodies that recognized both isoforms, any distinct changes in GAD65 protein could be masked by high levels of GAD67. Nevertheless, it is of note that SD had a significant effect on the combined GAD65/67 protein in the PF region only.

*In vivo* molecular changes, such as those detected in the present study, could be driven by circadian inputs orchestrated by the circadian clock, by a sleep-related increase in extracellular GABA levels, or by cellular activity related to the rest-activity cycle. Indeed, our data suggesting a negative feedback between the magnitude of GABAergic inhibition and both the availability of GABA_A_Rs and GABA synthesis may represent a special case of the broader concept proposed to explain the mechanisms of plasticity in the central nervous system in which a homeostatic component reactive to varying magnitudes of inhibitory and excitatory inputs plays a central role [99]–[101]. In particular, GABA_A_R subunit mRNA levels exhibit region- and subunit-specific changes in response to prolonged, systemic elevation of brain GABA levels [102]. Our results demonstrate that such processes occur on time scales of a single sleep-wake and circadian cycle, and that they utilize different components of the GABAergic system in two adjacent but functionally different regions of the posterior hypothalamus.

The effects of circadian time and amount of prior wakefulness on GABA_A_Rs in different regions of the posterior hypothalamus probably depend on both the nature and timing of the afferent inputs to cells in these two regions and on local interactions. We previously assessed the effects of stimulation or antagonism of GABA_A_Rs on GABA_A_R subunit and GAD mRNAs in the PF and DM regions of the posterior hypothalamus in slices *in vitro*, which effectively separated the studied regions from the main circadian pacemaker and eliminated any behavioral effects [44]. One important conclusion from that study was that pharmacological stimulation of all GABA receptors by muscimol resulted in a decline of mRNAs for all GABA_A_R subunits and GAD65 in both regions, but the effect was partially or fully antagonized by a selective GABA_A_R antagonist, gabazine, only in the PF region and for only selected mRNAs (α2, α3, β1, β3 and ε subunits, and GAD65). Furthermore, the mRNA level for the β1 subunit of the GABA_A_R was reduced following incubation of slices with muscimol after synaptic interactions were blocked by tetrodotoxin. These results demonstrated that local interactions occurring within the posterior hypothalamus and involving stimulation of GABA_A_Rs can provide negative feedback for the regulation of the strength of GABAergic inhibition in the PF region, whereas in the DM region inhibition mediated by non-GABA_A_R-dependent mechanisms has a more prominent role.

**Figure 8:**
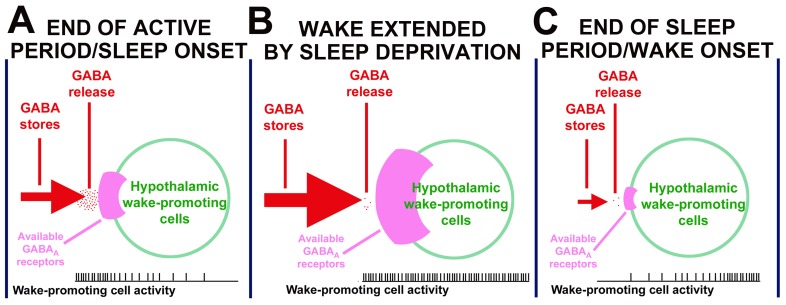
A model of the dynamic interactions involving GABA_A_ receptors, GABA stores and neuronal activity occurring within the wake-promoting region of the posterior hypothalamus supported by the findings from the present study. We propose that the availability of GABA_A_ receptors present in wake-promoting neurons increases with the duration of wakefulness, and so does the level of GABA in presynaptic terminals. A: when the combined strength of this potential inhibitory action is sufficiently high, sleepiness develops and sleep may ensue provided that it is not opposed by circadian mechanisms and/or excessive sensory stimulation. B: when wakefulness is extended by sleep deprivation, GABA_A_ receptors in wake-promoting neurons and GABA stores increase above the normal levels typical of the end of the active period. This may represent a neurochemical substrate of the increased drive for sleep. Under this condition, sleep occurs more readily and is deeper in the sense that it is more resistant to awakening stimuli. C: at the end of a complete rest/sleep period, the availability of GABA_A_ receptors on wake-promoting neurons is diminished due to their prolonged stimulation during sleep, and GABA stores are depleted. As a result, sleep-maintenance mechanisms are weakened and arousal followed by sustained wakefulness can readily occur. Our results suggest that GABA_A_ receptors containing β1, β3 and ε subunits located on wake-active posterior hypothalamic neurons are particularly important in this model because the levels of these subunits (mRNA and/or protein) vary with the pressure for sleep and show limited or no circadian time dependence.

A downregulation of GABA_A_Rs and reduced GABA synthesis following an increased stimulation of these receptors can provide a negative feedback that would facilitate termination of sleep and restoration of wakefulness. While such a mechanism can operate in many brain areas important for the regulation of sleep, here we provide specific evidence for the wake-promoting region of the posterior hypothalamus being one such a site and for a particular role of GABA_A_Rs that contain β1, β3 and ε subunits. Figure 8 presents a scheme of the dynamic interactions involving GABA_A_Rs, GABA levels and wake-active cells located in the PF region as they may contribute to the regulation of sleep in response to the prior period of wakefulness (homeostatically), as well as in response to circadian, ultradian and other behavioral cues. We propose that, in the PF region, GABA level increases in presynaptic terminals during prolonged wakefulness (through synthesis) and then declines during sleep (through depletion); and that the availability of GABA_A_Rs that contain β1, β3 and/or ε subunits on wake-active PF neurons increases when these cells are active and declines as a result of prolonged stimulation of these receptors. Accordingly, when wakefulness is extended by SD, more GABA accumulates in presynaptic terminals and additional GABA_A_Rs are synthesized in wake-active neurons (cf., Fig. 8A and 8B). This translates into an enhanced pressure for sleep (“sleepiness”) and makes sleep deeper and more difficult to terminate once it occurs. In contrast, at the end of the normal sleep period (Fig. 8C), GABA levels in presynaptic terminals are low and many of the previously available GABA_A_Rs have now been internalized and recycled. As a consequence, sleep can be terminated by relatively weak sensory or endogenous signals and, once awakening occurs, wakefulness is relatively easy to maintain. Our present data show that the interactions incorporated in the scheme in Fig. 8 are likely to take place in the PF region of the posterior hypothalamus, but they also may be applicable to other sleep-regulatory regions and other areas of the brain where recurring periods of cell activity occur within homeostatically delineated boundaries. Additional studies in animals with genetically altered GABA_A_Rs in distinct neuronal populations involved in the regulation of sleep should help further identify the cell types and connections that represent the key substrates of the brain “sleepiness signal.”

Our experiments with microperfusion of the PF region with a GABA_A_R antagonist at different circadian times and under varying pressure for sleep yielded results consistent with our data showing that a larger pool of GABA_A_Rs is available at the end of the active period and even more so after an extended period of wakefulness than when the need for sleep is partially discharged during the second half of the sleep period. Perfusion with a GABA_A_R antagonist reduced sleep most powerfully under the latter conditions. Considering that we used a relatively low concentration of the antagonist, our findings can be explained on the basis of the interaction between GABA levels and the availability of free and occupied GABA_A_Rs on wake active neurons, as schematically shown in Fig. 8. Alternatively, it is possible that signals proportional to the pressure for sleep are generated outside the PF region and that the varying effectiveness of GABA_A_R antagonism in the PF region on sleep is only secondary to sleep need-related processes occurring elsewhere. In support of the latter explanation, c-Fos data suggested that activity of anterior hypothalamic GABAergic neurons that play a key role in the maintenance of sleep is elevated following SD [103]. However, this mechanism is unlikely to underlie sleepiness before sleep is established. In another study, optogenetic stimulation of ORX neurons induced arousal less effectively following SD than under the normal level of sleep drive [104], which is similar to our results with BIC perfusions. However, the optogenetic approach would bypass any changes occurring presynaptically to ORX neurons. Our evidence that local, activity- and GABA_A_R-dependent [44], as well as the sleep need- and circadian time-dependent (this study), changes in GABA_A_Rs and GAD occur within the PF region shows that negative feedback suitable for a systemic regulation of sleep-wake states can operate in this region. Such a feedback probably converges on multiple wake-active neurons of this region, including ORX and non-ORX excitatory cells whose stimulation leads to multiple behavioral, cardiovascular, respiratory and metabolic signs of arousal [105]–[108].

Our finding that local perfusion of the PF region with a GABA_A_R antagonist had a particularly powerful REMS-reducing effect when it was applied during the second half of the sleep period (Fig. 6, central panel) deserves a special note. Under normal sleep conditions, the relative amount of REMS increases towards the latter part of the sleep period, but the neurochemical mechanisms underlying this phenomenon are unknown. We found that REMS was nearly abolished when the GABA_A_R antagonist was applied to the PF region during the second half of the sleep period despite the natural for this period increase of REMS amount. This suggests that time-dependent changes in inhibition mediated in the PF region by GABA_A_Rs importantly contribute to the progressively increasing amount of REMS with the duration of sleep.

### Conclusions

Our data demonstrate a potential for GABAergic transmission in the sleep-regulatory PF region of the posterior hypothalamus to vary with both the homeostatic and circadian regulation of sleep. We postulate that dynamically changing levels of GABA, GABA_A_R availability and levels of activity in wake-active neurons of the PF region interact in such a way that extended wakefulness leads to an increased potential for inhibition of wake-related neurons. The specific GABA_A_Rs that are involved in these processes are different from those in the DM hypothalamic region that is important for the regulation of various circadian time-dependent functions but not directly involved in the homeostatic regulation of sleep.
